# 

**Published:** 1893-10

**Authors:** 


					﻿ESTABLISHED IB5S.	SUBSCRIPTION.
ATLANTA
JVIEDIG Ah RUD SURGIGflh
JOURNAL.
neaSsek^4?vol. xX'L ( Atlanta, Ga., October, 1893. No. 8.
LUTHER B. GRANDY, M. D.,	M. B. HUTCHINS, M. I).,
MANAGING EDITOR.	BUSINESS MANAGER.
				

